# PLAGL1-IGF2 axis regulates osteogenesis of postnatal condyle development

**DOI:** 10.1038/s41368-025-00386-4

**Published:** 2025-09-25

**Authors:** Jinrui Sun, Jingyi Xu, Yue Xu, Yili Liu, Enhui Yao, Jiahui Du, Xinquan Jiang

**Affiliations:** 1 Department of Prosthodontics, Shanghai Ninth People’s Hospital, Shanghai Jiao Tong University School of Medicine, Shanghai, China; 2 College of Stomatology, Shanghai Jiao Tong University, Shanghai, China; 3 National Center for Stomatology, National Clinical Research Center for Oral Diseases, Shanghai Key Laboratory of Stomatology, Shanghai Research Institute of Stomatology, Shanghai Engineering Research Center of Advanced Dental Technology and Materials, Shanghai, China; 4 Shanghai Stomatological Hospital & School of Stomatology, Fudan University, Shanghai, China

**Keywords:** Bone development, Differentiation

## Abstract

The mandibular condyle is a critical growth center in craniofacial bone development, especially during postnatal stages. Postnatal condyle osteogenesis requires precise spatiotemporal coordination of growth factor signaling cascades and hierarchical gene regulatory networks. *Plagl1*, which encodes a zinc finger transcription factor, is a paternally expressed gene. We demonstrate that PLAGL1 is highly expressed in cranial neural crest cell (CNCC)-derived lineage cells in mouse condyles. Using the CNCC-derived lineage-specific *Plagl1* knockout mouse model, we evaluate the function of PLAGL1 during postnatal mouse condyle development. Our findings show that PLAGL1 contributes significantly to osteoblast differentiation, and its deficiency impairs osteogenic lineage differentiation, which consequently disrupts mandibular condyle development. Mechanistically, insulin-like growth factor 2 (IGF2) in complex with IGF-binding proteins (IGFBPs) has been identified as the principal PLAGL1 effector responsible for osteogenic regulation during postnatal condyle morphogenesis. *Plagl1* deficiency significantly downregulates the IGF2/IGFBP pathway, leading to disordered glucose metabolism, defective extracellular matrix organization, and impaired ossification. Exogenous IGF2 treatment rescues impaired osteoblast differentiation caused by *Plagl1* deficiency. In conclusion, the PLAGL1-IGF2 axis is a critical regulator of osteogenesis during mandibular condyle development.

## Introduction

The mandibular condyle, derived from cranial neural crest cells (CNCCs), serves as a primary growth site for the mandible and plays a crucial role in the overall formation, shape, and function of the jaw.^1^ Proper development of the mandibular condyle is essential for correct jaw alignment and function, including chewing and speaking. The mandibular condyle consists of mandibular condylar cartilage (MCC) and the subchondral bone.^2^ Recent evidence shows that multiple cell populations contribute to osteogenesis in the subchondral bone, including osteochondroprogenitors within the MCC, osteogenic progenitors beneath the cartilage, and those originating from the perichondrium and periosteum during condyle development.^3^

Postnatal condyle osteogenesis is tightly regulated by a complex interplay of growth factors and transcriptional networks.^4^ A precise combination of various growth factor-mediated signaling pathways, including insulin-like growth factors (IGF),^5^ transforming growth factor-beta (TGF-β),^6^ fibroblast growth factors,^7^ and Indian hedgehog pathways,^8^ is critical for ensuring proper cartilage and bone formation in the mouse condyle. In response to developmental signals, transcription factors (TFs) bind to cognate DNA motifs in enhancers or promoters of target genes, thereby controlling their spatial and temporal expression.^9^ TF networks are thought to determine the cell fate of stem and progenitor cells during the development of multiple tissues. Previous studies have shown that SOX9,^10^ RUNX2,^11^ and SP7^12^ regulate different stages of cartilage formation and bone development during mouse condyle development. These TFs interact with key signaling pathways, including PTHrP,^13^ and Notch signaling pathways,^14^ to precisely regulate osteogenesis. Dysregulation of these molecular pathways can lead to condyle dysplasia, resulting in clinical conditions such as abnormal occlusion, facial asymmetry, and micrognathia, which significantly affect both function and esthetics.^15^ Therefore, a deeper understanding of the transcriptional regulation mechanisms during condyle development could provide valuable insights for developing targeted therapeutic strategies for condyle dysplasia and related craniofacial abnormalities.

The imprinted gene network (IGN), which consists of hundreds of imprinted genes, plays a central role in embryonic development.^16–18^ Imprinted genes are expressed from only one allele, depending on whether the allele is inherited from the mother or father. This parent-of-origin-specific expression mainly occurs due to selective methylation of either the maternal or paternal allele.^19^ Notably, several imprinted genes encoding TFs have been identified as key regulators in developmental processes and gene regulation.^20^ Among these, *Plagl1*, a paternally expressed gene encoding a zinc finger TF, is critical for development in multiple tissues.^21^ It has been reported that PLAGL1 is involved in embryonic growth control, and its loss of function results in intrauterine growth restriction, with significant morphological alterations and impaired bone formation, particularly in the caudal vertebrae and ankle bones.^21–23^ In craniofacial tissue development, our previous study validated that PLAGL1 endows the chromatin remodeler ARID1A with its cell-type/spatial function during tooth root formation.^24^ A recent analysis of single-cell RNA sequencing data revealed that *Plagl1* is expressed in the mouse mandibular arches at embryo day 10.5, suggesting its potential role in mandibular development.^25^ Given its expression in mandibular mesenchymal cells and its established role in the CNCC-derived tooth root development, we hypothesize that PLAGL1 plays a critical role in osteogenesis during postnatal mandibular condyle development.

In this study, we first examined the expression pattern of PLAGL1. We found that from postnatal day (PN) 1.5 to PN 21.5, PLAGL1 is highly expressed in CNCC-derived lineage cells. To test our hypothesis, we generated CNCC-derived lineage-specific *Plagl1* knockout mice to evaluate its function during postnatal condyle development. Our results demonstrated that PLAGL1 contributes significantly to osteoblast differentiation, and *Plagl1* deficiency compromised the differentiation of osteogenic lineages, leading to impaired mandibular condyle development. Mechanistically, IGF2 has been identified as a critical downstream target of PLAGL1, mediating its role in osteogenesis during postnatal condyle development. *Plagl1* deficiency significantly downregulated the IGF2/insulin-like growth factor binding proteins (IGFBP) pathway, resulting in disordered glucose metabolism, defective extracellular matrix organization, and impaired ossification. Exogenous IGF2 treatment rescued impaired osteoblast differentiation following *Plagl1* deficiency. Collectively, our findings suggest that the PLAGL1-IGF2 axis plays a crucial role in regulating osteogenesis during mandibular condyle development.

## Results

### Expression Pattern of PLAGL1 in Mouse Condyles During Postnatal Development

**Fig. 1:**
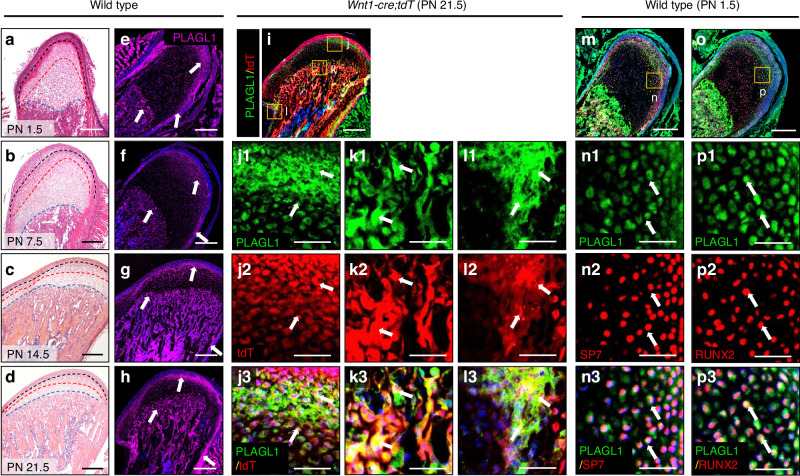
Expression pattern of PLAGL1 in mouse condyles during postnatal development. **a–d** Hematoxylin-eosin (H&E) staining images of the sagittal mandibular condyle in wild-type mice of postnatal day (PN) 1.5, PN 7.5, PN 14.5, PN 21.5. *n* = 3 per group. Black, red, and blue dot lines outline the boundary between fibrous/polymorphic layers, flat chondrocyte zone, and hypertrophic chondrocyte zone, respectively. Scale bars, 250 μm. **e–h** Immunofluorescence staining images of PLAGL1 (magenta) of the sagittal mandibular condyle in wild type mice of PN 1.5, PN 7.5, PN 14.5, PN 21.5. White arrows indicate positive signals. *n* = 3 per group. Scale bars, 250 μm. **i–l** Immunofluorescence staining images of PLAGL1 (green) and visualization of tdTdomato (tdT, red) of the mandibular condyle of a *Wnt1-cre;tdT* mouse of PN 21.5. *Wnt1+* lineage-derived cells show red signal. The yellow boxes represent the magnified regions in MCC (**j**), subchondral bone (**k**), and the perichondrium and periosteum (**l**). White arrows indicate *Wnt1+* lineage cells expressing PLAGL1. *n* = 3. Scale bar for i, 250 μm; scale bars for j1-j3, k1-k3, and l1-l3, 50 μm. **m-p** Immunofluorescence staining images of co-expression of PLAGL1 (green) with SP7 (red) (**m**, **n**) or RUNX2 (red) (**o**, **p**) in wild-type PN 1.5 mice. The yellow boxes (**n**, **p**) represent the magnified regions. White arrows denote PLAGL1+ cells co-expressing SP7 or RUNX2. *n* = 3. Scale bars for m and o, 250 μm; scale bars for n1-n3 and p1-p3, 50 μm

Postnatal mouse condyle development is classically divided into three stages: (1) chondrogenesis and proliferation, (2) hypertrophy and mineralization, and (3) ossification coupled with remodeling.^26,27^ The initial stage is chondrogenesis, during which chondrocytes proliferate within the condylar cartilage, establishing a growing cartilage template (Fig. 1a). In the next stage, chondrocytes undergo hypertrophy, secrete extracellular matrix, and initiate matrix mineralization (Fig. 1b). This transition of cartilage to a calcified state prepares it for replacement by bone. The mineralized cartilage is then replaced by bone tissue (Fig. 1c), followed by remodeling to achieve mature bone architecture (Fig. 1d). Hematoxylin-eosin (H&E) staining revealed the MCC’s characteristic stratified organization, consisting of four distinct zones: the fibrous and polymorphic tissue layers containing progenitor cells (above the black dashed line), the flattened zone (region between black and red dashed line), and hypertrophic zone (region between red and blue dashed line) (Fig. 1a–d). We observed a gradual decrease in MCC thickness along with progressive subchondral bone formation, consistent with typical condyle growth.

We further evaluated the dynamic expression pattern of PLAGL1 during key stages of postnatal condyle development at PN 1.5, PN 7.5, PN 14.5, and PN 21.5. Immunofluorescence staining demonstrated that PLAGL1 is expressed in the MCC, osteogenic progenitors in the subchondral bone, and the perichondrium and periosteum during postnatal condyle development (Fig. 1e–h). WNT1 is reported as a marker of neural crest cells.^28^ To further investigate the expression of PLAGL1 in CNCC-derived mesenchymal cells, *Wnt1-cre;tdTomato* (*tdT*) mice were generated for tracing CNCC-derived lineage cells. At PN 21.5, PLAGL1 was observed in CNCC-derived mesenchymal cells (tdT+ cells) within the MCC, subchondral bone, perichondrium, and periosteum, suggesting its involvement in both chondrogenic and osteogenic lineages (Fig. 1i–l). To further investigate the expression of PLAGL1 in osteochondroprogenitors in the MCC,^11^ we analyzed the co-localization of PLAGL1 with SP7 and RUNX2, two key TFs critical for endochondral ossification during postnatal condyle development (Fig. 1m–p). The co-expression of PLAGL1 with these osteogenic markers suggests its potential regulatory role in osteoblastic differentiation.

From this result, we speculate that PLAGL1 may play a regulatory role in promoting endochondral ossification during postnatal condyle development.

### *Plagl1* deficiency leads to impaired endochondral osteogenesis in mouse condyles

**Fig. 2:**
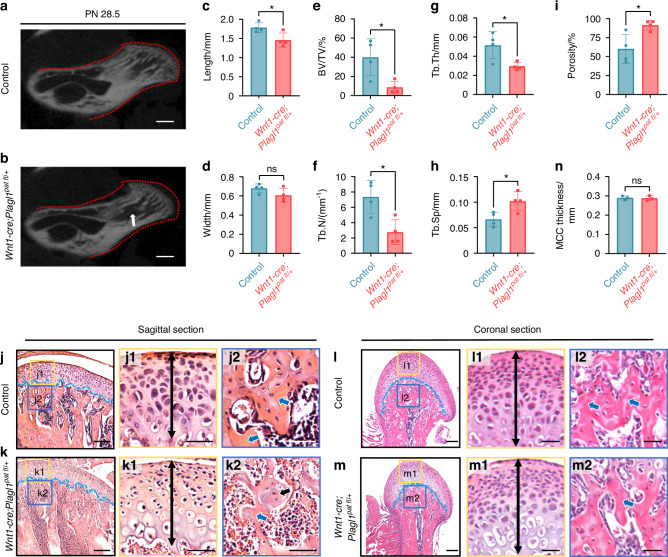
*Plagl1* deficiency leads to impaired endochondral osteogenesis in mouse condyles. **a**, **b** Micro-CT images of condyles of *Wnt1-Cre;Plagl1*^*pat fl/+*^ mice and control littermates at PN 28.5. White arrows show the enlarged bone marrow cavities and the red dashed lines show the normal morphology of condyle. Scale bars, 500 μm. **c–i** Measurements of condyle length, width, the ratio of bone volume to tissue volume (BV/TV), trabecular number (Tb.N), trabecular thickness (Tb.Th), trabecular separation (Tb.Sp) and porosity based on Micro-CT analysis. *n* = 4 per group. Unpaired two-tailed t-test was used for statistical analysis. H&E staining of the sagittal (**j**, **k**) and coronal (**l**, **m**) sections of the condyles of control and *Wnt1-Cre;Plagl1*^*pat fl/+*^ mice at PN 28.5. The blue lines show the demarcation between the articular cartilage and underlying subchondral bone. The yellow and blue boxes represent the magnified regions in MCC and subchondral bone (**j1**, **j2**, **k1**, **k2**, **l1**, **l2**, **m1**, **m2**). The black double-headed arrows show the thickness of MCC. The black arrow shows the remnant chondrocyte-like bone. The blue arrows show the subchondral bone. *n* = 3 per group. Scale bars for **j**–**m**, 125 μm; scale bars for j1, j2, k1, k2, l1, l2, m1, and m2, 50 μm. **n** Measurements for MCC thickness in control and *Wnt1-Cre;Plagl1*^*pat fl/+*^ mice. *n* = 3 per group. Unpaired two-tailed t-test was used for statistical analysis. The results are presented as the mean ± standard deviation (SD). *P < 0.05. ns, no significant difference

To investigate the potential role of PLAGL1 in postnatal condyle development, we compared the morphological phenotype of *Wnt1-Cre;Plagl1*^*pat fl/+*^ mice and their wild-type littermates at PN 28.5. Micro-CT analysis revealed a significant reduction in the length of the condyle and a reduced width of the condyle, although not significant, in *Plagl1*-deficient mice compared to controls (Fig. 2a–d). Additionally, we observed an osteoporotic phenotype in the subchondral bone of *Plagl1*-deficient mice compared to controls, characterized by a decreased bone volume to tissue volume ratio (BV/TV), trabecular number (Tb.N), and trabecular thickness (Tb.Th), along with an increased trabecular separation (Tb.Sp) and porosity (Fig. 2e–i). Furthermore, in *Plagl1*-deficient mice, we observed a significant reduction in mandibular size compared with controls (Fig. S1a). This was accompanied by decreased BV/TV and bone mineral density (BMD), as well as increased bone surface area to bone volume ratio (BS/BV) and porosity at the mandibular body-ramus junction (Fig. S1b). H&E staining revealed dense trabecular networks (blue arrow) with typical marrow cavity dimensions in control mice (Fig. 2j2). In contrast, *Plagl1*-deficient mice exhibited thinner trabeculae with reduced density (blue arrow, Fig. 2k2), chondrocyte-like osteoid remnants (black arrow, Fig. 2k2), and expanded marrow spaces, indicating a significant reduction in subchondral bone formation (Fig. 2j–m). However, the thickness of the MCC showed no obvious changes between *Wnt1-Cre;Plagl1*^*pat fl/+*^ mice and their wild-type littermates (Fig. 2n).

In conclusion, these findings suggest that PLAGL1 contributes significantly to postnatal condyle development, as *Plagl1* deficiency leads to subchondral bone dysplasia.

### *Plagl1* Deficiency leads to compromised osteoblastic differentiation in vivo and in vitro

**Fig. 3:**
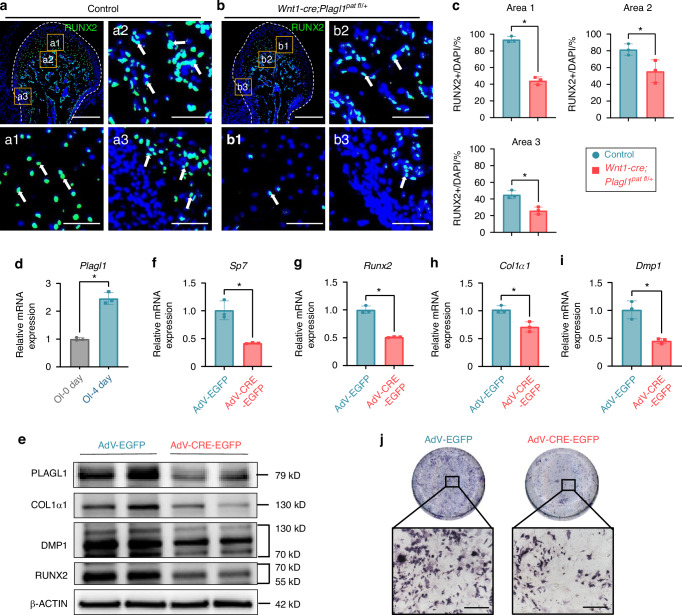
*Plagl1* deficiency leads to compromised osteoblastic differentiation in vivo and in vitro. **a**, **b** Immunofluorescence staining of RUNX2 (green) and DAPI (cyan) in condyles of control and *Wnt1-Cre;Plagl1*^*pat fl/+*^ mice at PN 28.5. The boxes (a1-a3, b1-b3) represent magnified regions. White arrows indicate the positive signals. *n* = 3 per group. Scale bars for a and b, 250 μm; scale bars for a1-a3, and b1-b3, 50 μm. **c** Analysis for the ratio of RUNX2+ cells in the MCC (area 1), subchondral bone (area 2), perichondrium and periosteum (area 3) of control and *Wnt1-Cre;Plagl1*^*pat fl/+*^ mice, *n* = 3 per group. Unpaired two-tailed t-test was used for statistical analysis. **d** qPCR analysis for the expression of *Plagl1* before osteogenic induction (OI-0 day) and after 4 days of osteogenic induction (OI-4 day). *n* = 3 per group. Unpaired two-tailed t-test was used for statistical analysis. **e** Western blot analysis of the expression of PLAGL1 and osteoblast-specific protein RUNX2, COL1α1 and DMP1 in adenovirus-enhanced green fluorescent protein (AdV-EGFP) and AdV-CRE-EGFP transfected condyle mesenchymal cells after osteogenic induction. **f**–**i** qPCR analysis for expression of osteoblast-specific gene *Sp7*, *Runx2*, *Col1α1* and *Dmp1* in AdV-EGFP and AdV-CRE-EGFP transfected condyle mesenchymal cells after osteogenic induction. *n* = 3 per group. Unpaired two-tailed t-test was used for statistical analysis. **j** ALP staining of AdV-EGFP and AdV-CRE-EGFP transfected condyle mesenchymal cells after 4 days of osteogenic induction. *n* = 3 per group. Scale bars, 200 μm. The results are presented as the mean ± standard deviation (SD). *P < 0.05

To investigate the cellular mechanisms underlying the phenotype observed in *Wnt1-Cre;Plagl1*^*pat fl/+*^ mice, we examined changes in osteoblastic bone formation at PN 28.5 following *Plagl1* deficiency. RUNX2 plays a key role in the growth and homeostasis of the MCC and subchondral bone formation in the mandibular condyle.^29^ Immunofluorescence staining and quantification showed that the number of RUNX2+ cells in the MCC, subchondral bone surface, perichondrium and periosteum were significantly reduced following *Plagl1* deficiency (Fig. 3a–c). We performed TRAP staining to assess the osteoclastic surface in subchondral bone of the condyles from control and *Wnt1-Cre;Plagl1*^*pat fl/+*^ mice. The results indicated that *Plagl1* deficiency did not significantly affect osteoclast activity in the subchondral bone of mouse condyles (Fig. S2). Taken together, these results suggest that *Plagl1* deletion in CNCCs impairs osteoblastic differentiation during endochondral bone formation in postnatal condyle development.

To further validate this finding, we isolated the mesenchymal cells from the mandibular condyle at PN 1.5. Using qPCR, we observed a significant increase in the expression of *Plagl1* during osteogenic induction (Fig. 3d). We then transfected mesenchymal cells from the mandibular condyle of *Plagl1*^*fl/fl*^ mice at PN 1.5 with Cre adenovirus (AdV-CRE-EGFP) to knock down *Plagl1* expression. The efficiency of PLAGL1 knockdown was confirmed by Western blot analysis, which showed a significant reduction of the expression of PLAGL1 at the protein level (Fig. 3e). Following osteogenic induction, *Plagl1* knockdown led to decreased expression of key osteogenic marker genes, including *Sp7*, *Runx2*, *Col1α1*, and *Dmp1* (Fig. 3f–i). ALP staining further demonstrated inhibited osteoblastic differentiation after 4 days of osteogenic induction in cells lacking *Plagl1* (Fig. 3j).

Taken together, these findings indicate that PLAGL1 contributes significantly to osteoblast differentiation during endochondral bone formation in postnatal condyle development.

### PLAGL1 transcription regulates osteoblastic differentiation-associated genes

To further elucidate the molecular mechanisms by which PLAGL1 regulates the osteoblastic differentiation of the mesenchymal cells from the mandibular condyle, we performed mRNA sequencing analysis to compare gene transcription profiles between the AdV-EGFP and AdV-CRE-EGFP groups before and after 4 days of osteoblastic induction.

**Fig. 4:**
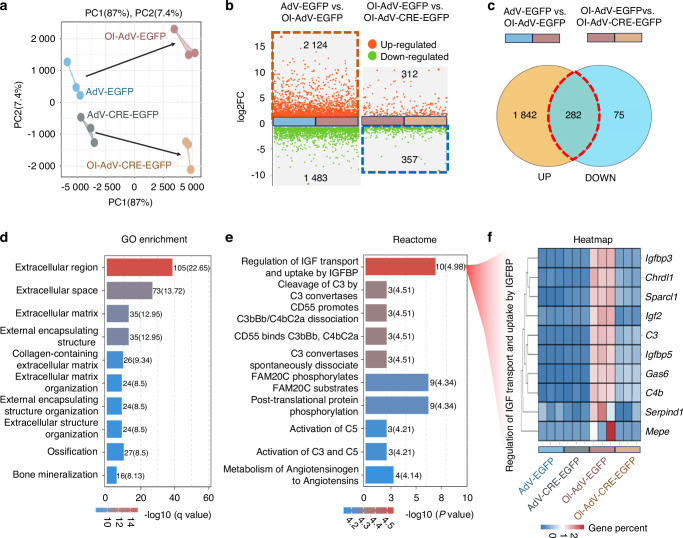
PLAGL1 transcription regulates osteoblastic differentiation-associated genes. **a** Principal component analysis (PCA) for gene expression patterns between AdV-EGFP and AdV-CRE-EGFP transfected condyle mesenchymal cells before and after osteogenic induction. *n* = 3 per group. **b** Genes differentially expressed between AdV-EGFP and AdV-CRE-EGFP transfected condyle mesenchymal cells before and after osteogenic induction. (|log2FoldChange | ≥ 1, FDR < 0.05). **c** Venn diagram for genes activated by osteogenic induction and inhibited by knockdown of *Plagl1*. **d** Top 10 gene ontology (GO) enriched biological processes in those genes activated by osteogenic induction while inhibited by knockdown of *Plagl1*. **e** Reactome enrichment analysis in genes those activated by osteogenic induction while inhibited by knockdown of *Plagl1*. **f** Heatmap for genes associated with the regulation of insulin-like growth factor (IGF) transport and uptake by insulin-like growth factor binding proteins (IGFBPs) between AdV-EGFP and AdV-CRE-EGFP transfected condyle mesenchymal cells before and after osteogenic induction. *n* = 3 per group

Principal component analysis (PCA) of the RNA sequencing data showed well-separated gene expression patterns between each group (Fig. 4a). As shown in Fig. 4b, osteogenic induction in AdV-EGFP cells resulted in 2 124 upregulated genes and 1 483 downregulated genes. Meanwhile, *Plagl1* knockdown led to 357 downregulated genes and 312 upregulated genes under osteogenic induction conditions (|log2FoldChange | ≥ 1, FDR < 0.05). To explore the molecular mechanisms by which PLAGL1 regulates osteoblast differentiation, we compared the 2 124 genes activated by osteogenic induction with the 357 genes promoted by PLAGL1 (Fig. 4c). Our analysis revealed 282 common genes that are induced during osteoblast differentiation but inhibited following *Plagl1* knockdown. This suggests that PLAGL1 plays a crucial role in promoting the expression of these osteogenic genes. Notably, approximately 80% of PLAGL1-promoted genes (282/357) are also activated during osteogenic induction, underscoring the critical role of PLAGL1 in osteoblast differentiation.

To further elucidate the pathways regulated by PLAGL1 during osteoblast differentiation, we performed gene ontology (GO) term enrichment analysis on the genes activated by osteogenic induction but inhibited following *Plagl1* knockdown (Fig. 4d). Among the top 10 enriched GO terms in biological processes, we identified several pathways related to extracellular matrix organization, ossification, and bone mineralization, highlighting PLAGL1’s role in regulating key processes involved in osteoblast differentiation. Notably, Reactome pathway analysis revealed that the regulation of IGF transport and uptake by IGFBP was the top enriched pathway (Fig. 4e). The IGF system is a complex network consisting of growth factors (IGF1 and IGF2), cell surface receptors (IGF1R and IGF2R), binding proteins (IGFBP1 to IGFBP6) and various other IGFBP-interacting molecules, all of which regulate and propagate IGF actions across multiple tissues.^30,31^ The heatmap of the top enriched pathway in the genes activated by osteogenic induction but inhibited following *Plagl1* knockdown demonstrated that the activation of these IGF/IGFBP pathway associated genes is dependent on PLAGL1 (Fig. 4f). Specifically, *Igf2*, *Igfbp3*, *Igfbp5-*encoded protein are the members of IGF/IGFBP axis involved in the development of mouse condyle.^32^ *Chrdl1* and *Gas6* encode binding proteins that modify the function of the IGF system action in bone development.^33,34^ For example, CHRDL1 directly binds to IGFBP3 and attenuates its degradation.^33^ GAS6 can abrogate IGF1-stimulated chondrocyte proliferation and stimulate differentiation of ATDC5 cells.^34^

### *Plagl1* deficiency leads to downregulated IGF2 and increased glucose metabolism

**Fig. 5:**
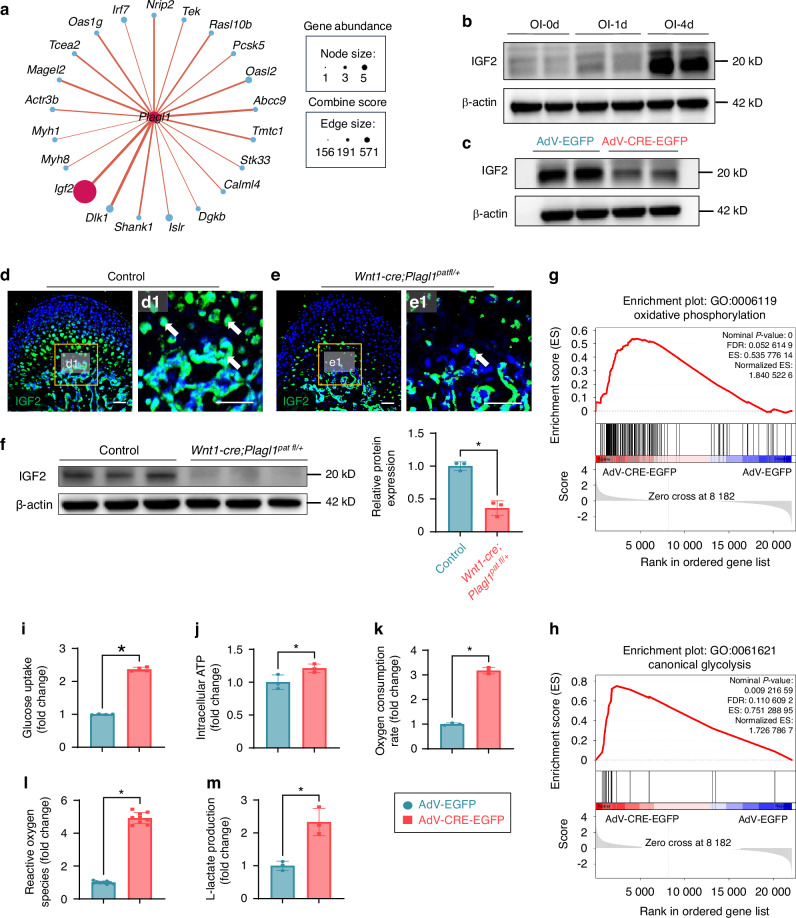
*Plagl1* deficiency leads to downregulated IGF2 and increased glucose metabolism. **a** Protein-protein interaction analysis for the relationship between PLAGL1 and genes activated by osteogenic induction while inhibited by knockdown of *Plagl1*. **b** Western blot analysis of the expression of IGF2 during osteogenic induction. **c** Western blot analysis of the expression of IGF2 after knockdown of *Plagl1*. **d**, **e** Immunofluorescence staining of IGF2 (green) and DAPI (cyan) in condyles of control and *Wnt1-Cre;Plagl1*^*pat fl/+*^ mice at PN 28.5. The boxes (**d1, e1**) show the magnified regions on the right. White arrows show the positive signals. *n* = 3 per group. Scale bars, 50 μm. **f** Western blot analysis of the expression of IGF2 in the subchondral bone after knockdown of *Plagl1*. Band intensities of IGF2 normalized to β-actin. *n* = 3 per group. Unpaired two-tailed t-test was used for statistical analysis. GSEA plots of GO term 0006119 (**g**) and 0061621 (**h**) between AdV-EGFP and AdV-CRE-EGFP transfected condyle mesenchymal cells after 4 days of osteogenic induction. *n* = 3 per group. Analysis of glucose uptake (**i**), intracellular ATP levels (**j**), oxygen consumption rate (**k**), reactive oxygen species (**l**), and L-lactate production (**m**) in AdV-EGFP and AdV-CRE-EGFP transfected condyle mesenchymal cells after 4 days of osteogenic induction. *n* = 4 per group for i. *n* = 3 per group for j, k, m. *n* = 9 per group for l. Unpaired two-tailed t-test was used for statistical analysis. The results are presented as the mean ± standard deviation (SD). *P < 0.05. ns, no significant difference

To further identify potential direct targets of PLAGL1, we performed protein-protein interaction analysis on the genes activated by osteogenic induction but inhibited following *Plagl1* knockdown. We identified IGF2 as a potential direct target of PLAGL1, with high expression abundance and a high prediction score (Fig. 5a). This suggests that PLAGL1 may directly activate IGF2, promoting extracellular matrix organization during osteoblastic differentiation. Previous studies have demonstrated that IGF2 is critical for osteoblast differentiation.^35–38^ Using Western blot analysis, we found that the expression of IGF2 significantly increased during osteoblast differentiation (Fig. 5b), but decreased after *Plagl1* knockdown (Fig. 5c). We identified IGF2 expression in the MCC, subchondral bone surface, perichondrium, and periosteum of the mandibular condyle in control mice at PN 28.5. However, there was a significant reduction in IGF2 expression in the *Wnt1-Cre;Plagl1*^*pat fl/+*^ mice compared to their littermate controls at PN 28.5 (Fig. 5d, e). The protein levels of IGF2 in the subchondral bone were downregulated in the *Wnt1-Cre;Plagl1*^*pat fl/+*^ mice compared to their littermate controls at PN 28.5 (Fig. 5f). These findings suggest that PLAGL1 promotes osteoblastic differentiation by activating the expression of IGF2.

Previous studies have shown that abnormal PLAGL1 function is responsible for transient neonatal diabetes mellitus (TNDM), a rare genetic disease that results from defective pancreas development, and suggested that PLAGL1 is associated with glucose metabolism.^39,40^ Additionally, IGF2 regulates bone growth through the modulation of glucose metabolism in chondrocytes.^41,42^ Based on these findings, we hypothesized that the deficiency of the PLAGL1-IGF2 signaling axis may lead to changes in glucose metabolism, impairing osteoblastic differentiation. Gene Set Enrichment Analysis (GSEA) showed that the oxidative phosphorylation and canonical glycolysis pathways were significantly upregulated after *Plagl1* deficiency compared to the control group (Fig. 5g, h). To investigate how glucose metabolism was changed after *Plagl1* deficiency, we measured overall glucose uptake (Fig. 5i), intracellular ATP production levels (Fig. 5j), oxygen consumption rate (Fig. 5k), reactive oxygen species (Fig. 5l), and L-lactate level (Fig. 5m) as indicators of cellular respiration and glycolysis. Compared to controls, *Plagl1*-deficient cells exhibited a significant increase in the above metabolic indicators. Collectively, these results suggest that *Plagl1* knockdown leads to an overall increase in glucose metabolism, including both oxidative phosphorylation and glycolysis.

### IGF2 administration partially rescues the osteoblastic differentiation of *Plagl1*-deficient Cells

**Fig. 6:**
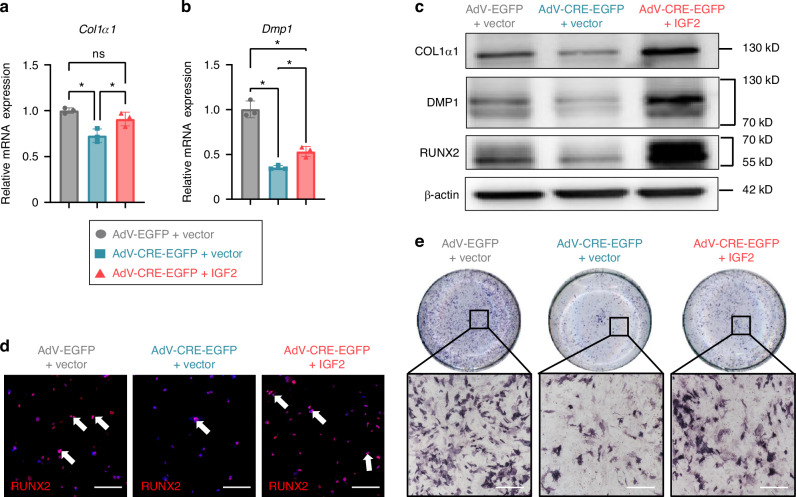
IGF2 administration partially rescues the osteoblastic differentiation of *Plagl1*-deficient cells. qPCR for the expression of osteoblast-linked gene *Col1α1* (**a**) and *Dmp1* (**b**) with the addition of IGF2 in AdV-EGFP and AdV-CRE-EGFP transfected condyle mesenchymal cells. *n* = 3 per group. One-way analysis of variance with Tukey’s post-hoc test was used for statistical analysis. **c** Western blot analysis of the expression of osteoblast-linked protein RUNX2, COl1α1, and DMP1 with the addition of IGF2 in AdV-EGFP and AdV-CRE-EGFP transfected condyle mesenchymal cells. **d** Immunofluorescence staining of rescued RUNX2 (red) and DAPI (cyan) in AdV-EGFP and AdV-CRE-EGFP transfected condyle mesenchymal cells with the addition of IGF2 or control vector. White arrows showed the positive signals. *n* = 3 per group. Scale bars, 250 μm. **e** ALP staining showing osteoblastic capacity in AdV-EGFP and AdV-CRE-EGFP transfected condyle mesenchymal cells with the addition of IGF2 or control vector. *n* = 3 per group. Scale bars, 200 μm. The results are presented as the mean ± standard deviation (SD). *P < 0.05. ns, no significant difference

To further confirm IGF2 is the principal PLAGL1 effector responsible for osteogenic regulation during postnatal condyle morphogenesis, we added exogenous IGF2 to *Plagl1*-deficient cells during osteogenic induction. The inhibited expression of osteogenic marker genes, including *Runx2*, *Col1α1*, and *Dmp1*, in *Plagl1*-deficient cells was rescued by exogenous IGF2 protein (Fig. 6a–d). Furthermore, ALP staining showed that the inhibited osteoblastic differentiation caused by *Plagl1* knockdown was rescued by exogenous IGF2 protein after 4 days of osteogenic induction (Fig. 6e). However, the addition of IGF2 did not fully rescue the ALP expression compared to the AdV-EGFP control group, we speculate that, in addition to IGF2, other affected downstream targets of PLAGL1 could contribute to the compromised osteoblast differentiation.

**Fig. 7:**
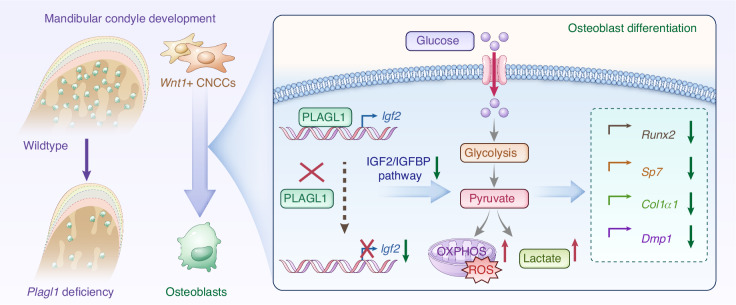
Schematic diagram of the PLAGL1-IGF2 axis in mandibular condylar development. Under physiological conditions, PLAGL1 activates *Igf2* expression to sustain the IGF2/IGFBP signaling pathway, thereby maintaining glucose metabolic homeostasis and promoting osteoblast differentiation through upregulation of key osteogenic markers of *Sp7*, *Runx2*, *Col1α1* and *Dmp1*. In contrast, *Plagl1* deficiency suppresses IGF2 signaling, triggering metabolic dysregulation characterized by aberrantly elevated oxidative phosphorylation and glycolysis, which collectively impair osteogenic differentiation and ultimately disrupt mandibular condylar morphogenesis

In conclusion, our results demonstrated that IGF2 is a critical downstream target of PLAGL1, mediating its role in osteogenesis during postnatal condyle development. *Plagl1* deficiency significantly downregulated the IGF2/IGFBP pathway, resulting in disordered glucose metabolism, defective extracellular matrix organization, and impaired ossification. Exogenous IGF2 treatment rescued impaired osteoblast differentiation following *Plagl1* deficiency. Collectively, our findings suggest that the PLAGL1-IGF2 axis plays a crucial role in regulating osteogenesis during mandibular condyle development (Fig. 7).

## Discussion

CNCCs display developmental plasticity and have the ability to differentiate into multiple cell types, including chondrocytes, osteoblasts, and odontoblasts.^43^ Osteochondroprogenitors exhibit a bipotential capability to differentiate into both chondrocytes and osteoblasts in the condyle.^44^ The proliferation and differentiation of these cells depend on the interaction of multiple signaling pathways and TFs. Disruptions in the normal expression patterns of TFs can lead to developmental disorders in the condyle. For example, in *Runx2*-deficient mice, the formation of MCC was blocked.^11^ The lack of *Sp7* in chondrocytes led to a significant increase in the thickness of the MCC due to the impediment of replacement of hypertrophic chondrocytes by endochondrally formed bone.^12^ In *Sox9*-null mice, mutant CNCC-derived cells shifted their chondrogenic cell fate to an osteoblast lineage, as indicated by increased expression of *Runx2*, *Sp7*, and *Col1α1*.^45^ In *Wnt1-Cre;Tgfbr2*^*fl/fl*^ mice, decreased expression of *Sox9* and enhanced expression of osteoblast markers such as *Runx2* and *Dlx5* resulted in bone formation without a cartilaginous intermediate.^6^

PLAGL1 is known for its antiproliferative activity and association with physiological cell cycle exit during contact inhibition, growth factor withdrawal, or cell differentiation.^46^ However, few studies have focused on its role in osteoblast differentiation in craniofacial tissue development. In this study, we identified PLAGL1 as a novel TF that contributes significantly to craniofacial osteoblast differentiation. The deficiency of *Plagl1* disrupted osteogenic lineage differentiation, resulting in impaired development of the mandibular condyle and significantly reduced bone mass in the mandibular bone. A deeper understanding of these developmental mechanisms is not only critical for developmental biology but also has significant implications for clinical dentistry in both humans and animals. First, PLAGL1 represents a promising therapeutic target for condylar hypoplasia. Local administration of agents that can demethylate the imprinted region of *Plagl1* in maternal allele, thereby increasing PLAGL1 expression, could potentially promote subchondral bone formation and facilitate condylar growth. Moreover, considering its role in intramembranous ossification, PLAGL1 could also serve as a target for promoting bone regeneration in jawbone defects. Additionally, our findings are particularly relevant for patients with TNDM who overexpress PLAGL1.^47^ These patients may require careful monitoring for potential condylar hyperplasia, as excessive PLAGL1 expression could lead to excessive bone formation. This understanding could guide preventive measures and inform treatment strategies for TNDM patients with craniofacial manifestations.

The IGN consists of hundreds of imprinted genes that play a central role in embryonic development.^16–18^ Interestingly, both PLAGL1 and its target, IGF2 in our study, belong to maternally imprinted genes. Our study identified an interaction between PLAGL1 and IGF2 in craniofacial bone tissue. We found that function loss of PLAGL1 led to significant downregulation of extracellular matrix organization and ossification, along with inhibition of the IGF/IGFBP pathway. The addition of exogenous IGF2 cytokine rescued the defective osteoblastic differentiation caused by *Plagl1* knockdown, further supporting our findings. These results suggested that PLAGL1-IGF2 axis promotes osteogenesis during condyle development, revealing their potential relationship in craniofacial tissue growth. Previous studies have linked IGF2 to the aberrant development of craniofacial and limb bones.^48^ A low level of IGF2 expression is associated with Silver-Russell syndrome, a human imprinting disorder characterized by postnatal short stature, distinctive facial features, and body asymmetry.^49^ In *Igf2*-null mice, an abnormal growth plate with an enlarged hypertrophic zone was observed, along with delayed secondary ossification and uncoordinated longitudinal and lateral growth in long bones.^41^ The PLAGL1-IGF2 axis has also been validated in Neuro2a neuroblastoma cell line, which is derived from postganglionic sympathetic neuroblasts that normally express *Plagl1* during development. Both gain and loss of PLAGL1 function elicit opposite regulation of imprinted genes such as *Cdkn1c*, *Igf2*, *H19*, and *Dlk1*.^21^ Therefore, we speculate that PLAGL1-IGF2 axis plays a conserved role in the development of multiple tissues. However, the addition of IGF2 does not fully rescue the ALP expression compared to the AdV-EGFP control group. As PLAGL1 has been reported to target 22% of the genes that constitute the IGN,^46^ there is still a significant knowledge gap regarding the function of these imprinted genes, particularly in their interactions with PLAGL1 during craniofacial bone development. We speculate that, in addition to IGF2, PLAGL1 may also regulate other critical genes in the IGN, which could also contribute to osteoblast differentiation—an area that remains to be further investigated.

It has been suggested that IGF2 plays an important role in the autocrine and paracrine-stimulated growth and remodeling of bone. In addition to osteoblast differentiation, IGF2 has also been implicated in promoting osteoclastogenesis.^50,51^ However, in the present study, the deficiency of *Plagl1* did not lead to any noticeable change in osteoclastic activity at the examined time point of PN 28.5. Although potential long-term effects cannot be entirely ruled out, the observed impairment in osteoblast differentiation strongly suggests that the deficiency of osteoblasts serves as the primary driver of osteogenic dysplasia during the early postnatal stages of condylar development. Further evaluation in adults should be considered, as bone remodeling processes could also be affected due to disrupted osteoblast-osteoclast interactions in the long term.

In addition, several imprinted genes have been found to regulate metabolic homeostasis in both infants and adults, some of which may coordinate growth with glucose-regulated metabolism.^52,53^ IGF2 exerts its effects by balancing glucose metabolism during cartilage development.^41,42^ PLAGL1, a candidate gene for TNDM, is also implicated in glucose homeostasis.^39,47^ In our study, we found that *Plagl1* knockdown reprogrammed glucose metabolic processes during osteoblast differentiation, as evidenced by the hyperactivation of both oxidative phosphorylation and glycolysis. It is indicated that glucose is a primary nutrient for bone growth and remodeling.^54,55^ Proper bone development requires balanced bioenergetic programs, allowing osteoblasts to adapt energy production and consumption to changing functional demands throughout their life cycle.^56^ While inhibiting oxidative phosphorylation and glycolysis impairs osteoblast differentiation,^57–60^ excessive glucose metabolism in growth plate chondrocytes similarly disrupts normal skeletal development.^41,42^ Therefore, an optimal level of glucose metabolism is necessary for normal bone development. Further studies are needed to explore how PLAGL1 balances glucose metabolism to promote skeletal development.

Furthermore, Reactome pathway analysis revealed a significant enrichment of complement-related processes among genes that were upregulated during osteogenic induction but downregulated following *Plagl1* knockdown. We speculate that the prominence of complement-related terms in Reactome analysis may stem from its detailed and specialized annotation of immune pathways, particularly those related to innate immunity. This likely explains why complement-related terms emerged prominently in Reactome but not in GO enrichment analysis. Moreover, emerging evidence suggests that the complement system may extend beyond its canonical immune defense roles to directly or indirectly regulate osteogenic differentiation. For instance, C3a and C5a receptors have been implicated in bone remodeling.^61,62^ Although our study did not experimentally validate PLAGL1’s function in the complement system, recent findings demonstrate that PLAGL1 overexpression induces cytoplasmic DNA accumulation, triggering cGAS/STING activation,^63^ which suggests a potential immunomodulatory role for PLAGL1 that might indirectly amplify complement-related transcriptional programs. This hypothesis warrants rigorous investigation in future studies to determine whether PLAGL1-complement crosstalk contributes to osteoblast differentiation.

In condyle development, hypertrophic chondrocytes, perichondrium, periosteum, and mesenchymal cells from bone marrow all contribute to the formation of osteoblasts.^3^ In our study, we found that *Plagl1* deficiency in condylar CNCC-derived mesenchymal lineage cells impairs the development of subchondral bone. Although we did not observe significant changes in the thickness of the MCC after the loss of *Plagl1*, the direct contribution of hypertrophic chondrocytes to osteoblast formation remains to be investigated. This should be studied separately from the contributions of the perichondrium, periosteum, and bone marrow. A deeper understanding of the integration of cartilage and bone formation is crucial for advancing regenerative medicine and developing effective therapeutic strategies that promote fibrocartilage and trabecular bone development.

In conclusion, understanding the regulatory mechanisms of condyle growth and development is crucial for addressing adolescent craniofacial bone malformations. While current research has provided valuable insights for clinical practice, further studies are needed to identify potential therapeutic targets and develop early intervention strategies.

## Materials and Methods

### Mice

*Wnt1-cre;Plagl1*^*pat fl/+*^ mice were generated by crossing *Wnt1-cre* female mice (Strain NO. 022501) from the Jackson Lab (Maine, USA) with *Plagl1*^*fl/fl*^ male mice (Strain NO. T038311) from GemPharmatech (Nanjing, China). *Plagl1*^*pat fl/+*^ mice were used for the control group in the cohort studies. TdTomato (Strain NO. 007909) mouse line^64^ was ordered from the Jackson Lab (Maine, USA). All mice were housed in pathogen-free conditions with constant ambient temperature (22 ± 2) °C and humidity (55% ± 10%), with an alternating 12-h light/dark cycle. Mice of both sexes were included in the analysis. All mice were euthanized by carbon dioxide overdose followed by cervical dislocation.

### Micro-CT analysis

Micro-CT analysis of fixed mandibular condyles was performed using a Skyscan 1176 (Bruker, Kontich, Belgium) at 60 kVp, 385 μA and a resolution of 9 μm. The obtained images were reconstructed with NRecon software (v1.7.1.0, Bruker, Kontich, Belgium). As previously described,^65^ a cubic region of interest (0.25 × 0.25 × 0.25 mm^3^) at the middle points of the center condyle was selected for examination of the longitudinal changes in subchondral bone for analyzing the ratio of bone volume to tissue volume (BV/TV), trabecular thickness (Tb.Th), trabecular separation (Tb.Sp), trabecular number (Tb.N) and total porosity. The length was measured as the anteroposterior diameter of condylar head, and the width was measured as the mediolateral diameter of condylar head. A 1 mm diameter circular region of full-thickness bone at the junction between the mandibular body and ramus was selected for analysis of BV/TV, the ratio of bone surface to bone volume (BS/BV), porosity and bone mineral density (BMD). All the analysis was proceeded by the CTAn software (v 1.16, Bruker, Kontich, Belgium).

### Histological analysis

The mandibular condyle from mice was dissected and fixed, followed by decalcification in 10% EDTA in PBS for 1–3 weeks, depending on the age of the samples. After decalcification, the samples were dehydrated using a graded series of ethanol and xylene and embedded in paraffin. The paraffin-embedded samples were sectioned at a thickness of 4 μm using a microtome (Leica). Hematoxylin and eosin (H&E) staining (Servicebio, G1005) and TRAP Staining (Sigma, 387 A) were performed according to the manufacturers’ protocols. Images were acquired using both a Nikon Eclipse Ti-U Microscope (Nikon, Japan) with NIS-Elements software (v4.5000.1117.0) and a Zeiss Axio Scope A1 Microscope (ZEISS, Germany) with DP2-TWAIN software (v3.0.0.6212).

### Immunofluorescence staining

Decalcified samples were dehydrated in a series of sucrose solutions and embedded in OCT compound (Tissue-Tek, Sakura). OCT-embedded samples were cryosectioned at 8 μm using a cryostat (Leica CM1850), followed by staining procedures. For immunofluorescence staining, the cryosections were first incubated in a blocking solution for one hour at room temperature. Subsequently, the sections were incubated with primary antibodies diluted in the blocking solution at 4 °C overnight. After washing the sections three times with PBST (PBS with 0.1% Tween 20), they were incubated with Alexa Fluor-conjugated secondary antibodies and counterstained with DAPI. The following primary antibodies were used: PLAGL1 antibody (Santa Cruz, sc166944, 1:50), RUNX2 antibody (Thermo Fisher Scientific, MA5-41185, 1:200), SP7 antibody (Abcam, ab22552, 1:200), and IGF2 antibody (Bioworld, BS40380, 1:100). The secondary antibodies included Donkey anti-Rabbit IgG (H + L) Highly Cross-Adsorbed Secondary Antibody, Alexa Fluor 594 (Thermo Fisher Scientific, A-21207, 1:200), Goat anti-Rabbit IgG (H + L) Cross-Adsorbed Secondary Antibody, Alexa Fluor 488 (Thermo Fisher Scientific, A-11008, 1:200) and Goat anti-Mouse IgG (H + L) Cross-Adsorbed Secondary Antibody, Alexa Fluor 488 (Thermo Fisher Scientific, A-11001, 1:200).

### Cell culture and osteoblast differentiation

The mesenchymal cells were harvested from the mandibular condyle of *Plagl1*^*fl/fl*^ mice at PN 1.5. The dissected condyles were cut into small pieces and plated. The cells were cultured in α-minimum essential medium (α-MEM, BasalMedia Co., Ltd, L510KJ) containing 10% fetal bovine serum (FBS; Cyagen Biosciences, OriCell® FBSAD-01011-500) and 1% penicillin-streptomycin (Gibco, 15140122) overnight. Passage 2 mesenchymal cells were used for further experiments.

For osteoblast differentiation, cells were cultured in a complete medium supplemented with 50 μg/mL ascorbic acid (Sigma-Aldrich, A4544), 5 mmol/L β-glycerophosphate (Sigma-Aldrich, G9422), and 0.1 μmol/L dexamethasone (Sigma-Aldrich, D4902). The medium was changed every 2 days until the indicated time points. To confirm that IGF2 is a downstream target of PLAGL1 during osteoblastic differentiation, we added recombinant human IGF2 (Novoprotein, CF61) at 250 ng/mL in *Plagl1*-deficient cells during osteogenic induction.

### Alkaline Phosphatase (ALP) staining

ALP staining was performed to examine the osteogenic ability of mesenchymal cells from the mandibular condyle. Briefly, mesenchymal cells from the mandibular condyle were induced to undergo osteogenic differentiation to perform ALP staining. At the corresponding time point, the cells were washed twice with PBS and then fixed with 4% paraformaldehyde for 10 minutes, followed by two additional washes with PBS. Then, cells were incubated with an ALP staining solution (Beyotime, C3206) for 30 minutes. After washing twice with PBS, the stained cells were visualized and photographed using a Nikon Eclipse Ti-U Microscope (Nikon) with NIS-Elements software (v4.5000.1117.0).

### Adenovirus transfection

For adenovirus (AdV) transfection, 2 × 10^5^ cells/mL were seeded in 6-well or 12-well plates in a complete culture medium and incubated overnight. AdV-CRE-EGFP and its control adenovirus, AdV-EGFP, were purchased from Hanbio Biotechnology Co. Ltd (Shanghai, China). Before transfection, the culture medium was replaced with fresh medium without penicillin-streptomycin, and the volume was reduced to half. The cells were then infected with either AdV-CRE-EGFP or control AdV-EGFP at a multiplicity of infection of 100. After 4 hours, the remaining half of the culture medium was added to each well. After 12–16 hours, the medium was replaced with a fresh complete culture medium. After 24 hours, the transfected cells were induced with osteogenic differentiation medium for further analysis.

### Quantitative reverse transcription PCR

Total RNA was extracted from cells using TRIzol reagent (Sigma, T9424), and reverse transcription was performed using the PrimeScript RT Master Kit (TakaRa Bio Inc., RR036A). Quantitative reverse transcription PCR (qPCR) was conducted using a Roche LightCycler 480 system (v1.5.1.74) with SYBR Green Supermix (Yeasen, 11201ES). The relative expression levels of each mRNA transcript were calculated using the 2^-ΔΔCt^ method, with β-actin serving as the internal control for normalization. The primers used for qPCR are listed in Table S1.

### Western blotting

For western blot analysis, cells were collected, and total protein was extracted and homogenized in RIPA buffer (Thermo Fisher Scientific, 89900) supplemented with protease inhibitor (Thermo Fisher Scientific, A32959). The extracted proteins were separated by SDS-PAGE using 10% acrylamide gels (ZHHC, PE008) and transferred onto PVDF membranes (Millipore, IPVH00005). The membranes were blocked in 5% skimmed milk for 1 hour at room temperature and incubated with the following primary antibodies overnight at 4 °C: PLAGL1 antibody (Novus, NBP3-41420, 1:500), RUNX2 antibody (Thermo Fisher Scientific, MA5-41185, 1:1 000), DMP1 antibody (Affinity, DF8825, 1:1 000), COL1α1 antibody (Servicebio, GB115707, 1:500), IGF2 antibody (Bioworld, BS40380, 1:1 000), and β-actin antibody (Bioworld, BS67001, 1:2000). After washing the membranes three times with 0.01% TBST, they were incubated with HRP-conjugated secondary antibodies: Mouse IgG HRP-conjugated antibody (R&D, HAF007, 1:2 000) and Rabbit IgG HRP-conjugated antibody (R&D, HAF008, 1:2 000). Visualization of the signals was carried out using BeyoECL Moon (Beyotime, P0018FM), and the signals were detected using the UVITEC Alliance system (v16.0.3.0). Band intensities were quantified using ImageJ (v1.54).

### Glucose metabolism assays

To evaluate glucose metabolism, mouse condyle mesenchymal cells transfected with AdV-EGFP and AdV-CRE-EGFP were cultured in osteogenic differentiation medium for four days. For glucose uptake, cells were treated with glucose-free DMEM (BasalMedia, L160KJ) containing 200 μmol/L 2-NBDG (Beyotime, S0561S). The uptake of 2-NBDG was measured 15 minutes later using a Tecan Spark 10 mol/L plate reader (Switzerland). To measure the oxygen consumption rate, cells were plated in black 96-well cell culture plates with clear bottoms (Beyotime, FCP965) at a density of 70 000 cells per well and measured using the Oxygen Consumption Assay Kit (Bestbio, BB-48211). Additionally, reactive oxygen species levels were monitored using the ROS Assay Kit (Beyotime, S0033S), with readings taken on the plate reader. For intracellular ATP levels, cells were assayed using the Enhanced ATP Assay Kit (Beyotime, S0027), and luminescence was detected using the plate reader. To quantify L-lactate production, cells were extracted using the L-lactate Assay Kit (Beyotime, S0208S), and absorbance was measured in the plate reader. In all assays, the total cell numbers based on BCA assay (Yeasen, 20201ES90) or cell counting were used for normalization.

### RNA sequencing

For RNA-sequencing (RNA-seq) analysis, libraries were prepared using NEBNext Ultra II RNA Library Prep Kit and then sequenced on Illumina NovaSeq platform. Raw reads were filtered using Cutadapt (v1.15) and aligned with the GRCm39 genome using HISAT2 (v2.0.5). Read count values for each gene were calculated using HTSeq (v0.9.1) and normalized to fragments per kilobase of transcript per million mapped reads. Principal component analysis was performed with R package gmodels (http://www.r-project.org/). Differentially expressed genes (DEGs) were identified using DESeq2 (|log2FoldChange | ≥ 1, FDR < 0.05). DEGs were then mapped to Gene Ontology (GO) terms in the Gene Ontology database (http://www.geneontology.org/), with gene numbers calculated for each term. GO term enrichment analysis was performed using a hypergeometric test to compare DEGs with the genome background.^66^ Additionally, Reactome enrichment analysis^67,68^ was conducted to identify significantly enriched pathways (FDR ≤ 0.05).

### Protein-protein interaction analysis

Protein-protein interaction network for the identified DEGs was identified using String (v10)^69^, which determined genes as nodes and interactions as lines in a network. The network file was visualized using Cytoscape software (v3.7.1)^70^ to present core and hub genes in the biological interaction network.

### Statistical analysis

All statistical analyses were conducted using GraphPad Prism (v10.1.2) software. The results are presented as the mean ± standard deviation (SD). For comparisons between the two groups, an unpaired two-tailed t-test was used. For comparisons between multiple groups, one-way analysis of variance (ANOVA) with Tukey’s post-hoc test was used. Statistical significance was defined as a probability (*P*) value < 0.05. The sample sizes (n), P-values, and specific statistical tests performed for each experiment are described in the corresponding figure legends.

## Supplementary information

Supplementary data

## Data Availability

The bulk RNA-seq data generated in this study have been deposited in the Gene Expression Omnibus database under accession code GSE279365. GRCm39 genome is referenced in this study [http://asia.ensembl.org/Mus_musculus/Info/Index]. The original contributions presented in the study are included in the article/Supplementary Material, further inquiries can be directed to the corresponding authors.

## References

[1] Yuan, Y. & Chai, Y. Regulatory mechanisms of jaw bone and tooth development. *Curr. Top. Dev. Biol.* **133**, 91–118 (2019). 30902260 10.1016/bs.ctdb.2018.12.013 PMC6986348

[2] Rogers, A. W., Cisewski, S. E. & Kern, C. B. The zonal architecture of the mandibular condyle requires ADAMTS5. *J. Dent. Res.* **97**, 1383–1390 (2018). 29879379 10.1177/0022034518777751 PMC6199677

[3] Hinton, R. J., Jing, Y., Jing, J. & Feng, J. Q. Roles of Chondrocytes in endochondral bone formation and fracture repair. *J. Dent. Res.* **96**, 23–30 (2017). 27664203 10.1177/0022034516668321 PMC5347428

[4] Stocum, D. L. & Roberts, W. E. Part I: Development and physiology of the temporomandibular joint. *Curr. Osteoporos. Rep.* **16**, 360–368 (2018). 29948821 10.1007/s11914-018-0447-7

[5] Bi, R. et al. Igf1 regulates fibrocartilage stem cells, cartilage growth, and homeostasis in the temporomandibular joint of mice. *J. Bone Miner. Res.* **38**, 556–567 (2023). 36722289 10.1002/jbmr.4782

[6] Oka, K. et al. The role of TGF-β signaling in regulating chondrogenesis and osteogenesis during mandibular development. *Dev. Biol.* **303**, 391–404 (2007). 17204263 10.1016/j.ydbio.2006.11.025 PMC2074881

[7] Molténi, A., Modrowski, D., Hott, M. & Marie, P. J. Differential expression of fibroblast growth factor receptor-1, -2, and -3 and syndecan-1, -2, and -4 in neonatal rat mandibular condyle and calvaria during osteogenic differentiation in vitro. *Bone* **24**, 337–347 (1999). 10221546 10.1016/s8756-3282(98)00191-4

[8] Kurio, N. et al. Roles of Ihh signaling in chondroprogenitor function in postnatal condylar cartilage. *Matrix Biol.* **67**, 15–31 (2018). 29447948 10.1016/j.matbio.2018.02.011 PMC5910228

[9] Lambert, S. A. et al. The human transcription factors. *Cell* **172**, 650–665 (2018). 29425488 10.1016/j.cell.2018.01.029 PMC12908702

[10] Kitamura, A. et al. Downregulation of SOX9 expression in developing entheses adjacent to intramembranous bone. *PLoS One* **19**, e0301080 (2024). 38728328 10.1371/journal.pone.0301080 PMC11086909

[11] Shibata, S. et al. Runx2-deficient mice lack mandibular condylar cartilage and have deformed Meckel’s cartilage. *Anat. Embryol.* **208**, 273–280 (2004). 10.1007/s00429-004-0393-2 15156401

[12] Jing, J. et al. Osterix couples chondrogenesis and osteogenesis in post-natal condylar growth. *J. Dent. Res.* **93**, 1014–1021 (2014). 25192899 10.1177/0022034514549379 PMC4212325

[13] Tsutsumi-Arai, C. et al. A PTHrP gradient drives mandibular condylar chondrogenesis via Runx2. *J. Dent. Res.* **103**, 91–100 (2024). 38058151 10.1177/00220345231208175 PMC10734211

[14] Ruscitto, A. et al. Notch regulates fibrocartilage stem cell fate and is upregulated in inflammatory TMJ arthritis. *J. Dent. Res.* **99**, 1174–1181 (2020). 32442041 10.1177/0022034520924656 PMC7443994

[15] Fabik, J., Psutkova, V. & Machon, O. The Mandibular and Hyoid Arches-from molecular patterning to shaping bone and cartilage. *Int. J. Mol. Sci.* **22**, 7529 (2021). 34299147 10.3390/ijms22147529 PMC8303155

[16] Thamban, T., Agarwaal, V. & Khosla, S. Role of genomic imprinting in mammalian development. *J. Biosci.* **45**, 20 (2020). 31965998

[17] Al Adhami, H. et al. A systems-level approach to parental genomic imprinting: the imprinted gene network includes extracellular matrix genes and regulates cell cycle exit and differentiation. *Genome Res.* **25**, 353–367 (2015). 25614607 10.1101/gr.175919.114 PMC4352888

[18] Matoba, S. et al. Noncanonical imprinting sustains embryonic development and restrains placental overgrowth. *Genes Dev.* **36**, 483–494 (2022). 35483741 10.1101/gad.349390.122 PMC9067403

[19] Hanna, C. W. & Kelsey, G. Features and mechanisms of canonical and noncanonical genomic imprinting. *Genes Dev.* **35**, 821–834 (2021). 34074696 10.1101/gad.348422.121 PMC8168557

[20] Hamed, M., Ismael, S., Paulsen, M. & Helms, V. Cellular functions of genetically imprinted genes in human and mouse as annotated in the gene ontology. *PLoS One* **7**, e50285 (2012). 23226257 10.1371/journal.pone.0050285 PMC3511506

[21] Varrault, A. et al. Zac1 regulates an imprinted gene network critically involved in the control of embryonic growth. *Dev. Cell.* **11**, 711–722 (2006). 17084362 10.1016/j.devcel.2006.09.003

[22] Piras, G. et al. Zac1 (Lot1), a potential tumor suppressor gene, and the gene for epsilon-sarcoglycan are maternally imprinted genes: identification by a subtractive screen of novel uniparental fibroblast lines. *Mol. Cell Biol.* **20**, 3308–3315 (2000). 10757814 10.1128/mcb.20.9.3308-3315.2000 PMC85624

[23] Valente, T. & Auladell, C. Expression pattern of Zac1 mouse gene, a new zinc-finger protein that regulates apoptosis and cellular cycle arrest, in both adult brain and along development. *Mech. Dev.* **108**, 207–211 (2001). 11578877 10.1016/s0925-4773(01)00492-0

[24] Du, J. et al. Arid1a-Plagl1-Hh signaling is indispensable for differentiation-associated cell cycle arrest of tooth root progenitors. *Cell Rep.* **35**, 108964 (2021). 33826897 10.1016/j.celrep.2021.108964 PMC8132592

[25] Xu, J. et al. Hedgehog signaling patterns the oral-aboral axis of the mandibular arch. *Elife* **8**, e40315 (2019). 30638444 10.7554/eLife.40315 PMC6347453

[26] Shen, G. & Darendeliler, M. A. The adaptive remodeling of condylar cartilage-a transition from chondrogenesis to osteogenesis. *J. Dent. Res.* **84**, 691–699 (2005). 16040724 10.1177/154405910508400802

[27] Silbermann, M. & Livne, E. Skeletal changes in the condylar cartilage of the neonate mouse mandible. *Biol. Neonate.* **35**, 95–105 (1979). 420892 10.1159/000241159

[28] Chai, Y. et al. Fate of the mammalian cranial neural crest during tooth and mandibular morphogenesis. *Development* **127**, 1671–1679 (2000). 10725243 10.1242/dev.127.8.1671

[29] Liao, L. et al. Deletion of Runx2 in condylar chondrocytes disrupts TMJ tissue homeostasis. *J. Cell Physiol.* **234**, 3436–3444 (2019). 30387127 10.1002/jcp.26761 PMC6318053

[30] LeRoith, D., Holly, J. M. P. & Forbes, B. E. Insulin-like growth factors: Ligands, binding proteins, and receptors. *Mol. Metab.* **52**, 101245 (2021). 33962049 10.1016/j.molmet.2021.101245 PMC8513159

[31] Denley, A., Cosgrove, L. J., Booker, G. W., Wallace, J. C. & Forbes, B. E. Molecular interactions of the IGF system. *Cytokine Growth Factor Rev.* **16**, 421–439 (2005). 15936977 10.1016/j.cytogfr.2005.04.004

[32] Shibata, S., Fukuoka, H., Sato, R., Abe, T. & Suzuki, Y. An in situ hybridization study of the insulin-like growth factor system in developing condylar cartilage of the fetal mouse mandible. *Eur. J. Histochem.* **56**, e23 (2012). 22688304 10.4081/ejh.2012.e23 PMC3428972

[33] Sun, H. et al. Chordin Like-1 Regulates Osteoblast and Adipocyte differentiation through stabilizing insulin-like growth factor binding Protein 3. *Stem Cells* **41**, 400–414 (2023). 36682027 10.1093/stmcls/sxad009

[34] Hutchison, M. R., Bassett, M. H. & White, P. C. SCF, BDNF, and Gas6 are regulators of growth plate chondrocyte proliferation and differentiation. *Mol. Endocrinol.* **24**, 193–203 (2010). 19897599 10.1210/me.2009-0228 PMC2802903

[35] Hamidouche, Z., Fromigué, O., Ringe, J., Häupl, T. & Marie, P. J. Crosstalks between integrin alpha 5 and IGF2/IGFBP2 signalling trigger human bone marrow-derived mesenchymal stromal osteogenic differentiation. *BMC Cell Biol.* **11**, 44 (2010). 20573191 10.1186/1471-2121-11-44 PMC2901205

[36] Chen, L. et al. Insulin-like growth factor 2 (IGF-2) potentiates BMP-9-induced osteogenic differentiation and bone formation. *J. Bone Miner. Res.* **25**, 2447–2459 (2010). 20499340 10.1002/jbmr.133 PMC3179288

[37] Hardouin, S. N., Guo, R., Romeo, P. H., Nagy, A. & Aubin, J. E. Impaired mesenchymal stem cell differentiation and osteoclastogenesis in mice deficient for Igf2-P2 transcripts. *Development* **138**, 203–213 (2011). 21148188 10.1242/dev.054916

[38] Fanganiello, R. D. et al. Increased In Vitro Osteopotential in SHED associated with higher IGF2 expression when compared with hASCs. *Stem Cell Rev. Rep.* **11**, 635–644 (2015). 25931278 10.1007/s12015-015-9592-x

[39] Ma, D. et al. Impaired glucose homeostasis in transgenic mice expressing the human transient neonatal diabetes mellitus locus, TNDM. *J. Clin. Invest.* **114**, 339–348 (2004). 15286800 10.1172/JCI19876 PMC484972

[40] Varrault, A. et al. Characterization of the methylation-sensitive promoter of the imprinted ZAC gene supports its role in transient neonatal diabetes mellitus. *J. Biol. Chem.* **276**, 18653–18656 (2001). 11297535 10.1074/jbc.C100095200

[41] Uchimura, T. et al. An essential role for IGF2 in cartilage development and glucose metabolism during postnatal long bone growth. *Development* **144**, 3533–3546 (2017). 28974642 10.1242/dev.155598 PMC5665487

[42] Hollander, J. M. et al. A critical bioenergetic switch is regulated by IGF2 during murine cartilage development. *Commun. Biol.* **5**, 1230 (2022). 36369360 10.1038/s42003-022-04156-4 PMC9652369

[43] Bronner-Fraser, M. Origins and developmental potential of the neural crest. *Exp. Cell Res.* **218**, 405–417 (1995). 7796877 10.1006/excr.1995.1173

[44] Silbermann, M. et al. Chondroid bone arises from mesenchymal stem cells in organ culture of mandibular condyles. *J. Craniofac. Genet. Dev. Biol.* **7**, 59–79 (1987). 3597722

[45] Mori-Akiyama, Y., Akiyama, H., Rowitch, D. H. & de Crombrugghe, B. Sox9 is required for determination of the chondrogenic cell lineage in the cranial neural crest. *Proc. Natl. Acad. Sci. Usa.* **100**, 9360–9365 (2003). 12878728 10.1073/pnas.1631288100 PMC170923

[46] Varrault, A. et al. Identification of Plagl1/Zac1 binding sites and target genes establishes its role in the regulation of extracellular matrix genes and the imprinted gene network. *Nucleic Acids Res* **45**, 10466–10480 (2017). 28985358 10.1093/nar/gkx672 PMC5737700

[47] Kamiya, M. et al. The cell cycle control gene ZAC/PLAGL1 is imprinted-a strong candidate gene for transient neonatal diabetes. *Hum. Mol. Genet.* **9**, 453–460 (2000). 10655556 10.1093/hmg/9.3.453

[48] Sélénou, C., Brioude, F., Giabicani, E., Sobrier, M. L. & Netchine, I. IGF2: Development, Genetic and Epigenetic Abnormalities. *Cells* **11**, 1886 (2022). 35741015 10.3390/cells11121886 PMC9221339

[49] Loid, P. et al. Case report: A novel de novo IGF2 missense variant in a Finnish patient with Silver-Russell syndrome. *Front. Pediatr.* **10**, 969881 (2022). 36268036 10.3389/fped.2022.969881 PMC9578642

[50] Hill, P. A., Reynolds, J. J. & Meikle, M. C. Osteoblasts mediate insulin-like growth factor-I and -II stimulation of osteoclast formation and function. *Endocrinology* **136**, 124–131 (1995). 7828521 10.1210/endo.136.1.7828521

[51] Kondo, T. et al. Insulin-like growth factor 2 promotes osteoclastogenesis increasing inflammatory cytokine levels under hypoxia. *J. Pharmacol. Sci.* **149**, 93–99 (2022). 35641033 10.1016/j.jphs.2022.03.007

[52] Smith, F. M., Garfield, A. S. & Ward, A. Regulation of growth and metabolism by imprinted genes. *Cytogenet. Genome Res.* **113**, 279–291 (2006). 16575191 10.1159/000090843

[53] Peters, J. The role of genomic imprinting in biology and disease: an expanding view. *Nat. Rev. Genet.* **15**, 517–530 (2014). 24958438 10.1038/nrg3766

[54] Lecka-Czernik, B. & Rosen, C. J. Energy excess, glucose utilization, and skeletal remodeling: new insights. *J. Bone Miner. Res.* **30**, 1356–1361 (2015). 26094610 10.1002/jbmr.2574

[55] Karsenty, G. & Khosla, S. The crosstalk between bone remodeling and energy metabolism: A translational perspective. *Cell Metab.* **34**, 805–817 (2022). 35545088 10.1016/j.cmet.2022.04.010 PMC9535690

[56] Riddle, R. C. & Clemens, T. L. Bone Cell bioenergetics and skeletal energy homeostasis. *Physiol. Rev.* **97**, 667–698 (2017). 28202599 10.1152/physrev.00022.2016 PMC5539406

[57] Lee, S. Y. & Long, F. Notch signaling suppresses glucose metabolism in mesenchymal progenitors to restrict osteoblast differentiation. *J. Clin. Invest.* **128**, 5573–5586 (2018). 30284985 10.1172/JCI96221 PMC6264656

[58] Esen, E. et al. WNT-LRP5 signaling induces Warburg effect through mTORC2 activation during osteoblast differentiation. *Cell Metab.* **17**, 745–755 (2013). 23623748 10.1016/j.cmet.2013.03.017 PMC3653292

[59] Gao, J. et al. SIRT3/SOD2 maintains osteoblast differentiation and bone formation by regulating mitochondrial stress. *Cell Death Differ.* **25**, 229–240 (2018). 28914882 10.1038/cdd.2017.144 PMC5762839

[60] Dobson, P. F. et al. Mitochondrial dysfunction impairs osteogenesis, increases osteoclast activity, and accelerates age related bone loss. *Sci. Rep.* **10**, 11643 (2020). 32669663 10.1038/s41598-020-68566-2 PMC7363892

[61] Kuhn, M. B. et al. C3a-C3aR signaling is a novel modulator of skeletal homeostasis. *Bone Rep.* **18**, 101662 (2023). 36860797 10.1016/j.bonr.2023.101662 PMC9969257

[62] Matsuoka, K., Park, K. A., Ito, M., Ikeda, K. & Takeshita, S. Osteoclast-derived complement component 3a stimulates osteoblast differentiation. *J. Bone Miner. Res.* **29**, 1522–1530 (2014). 24470120 10.1002/jbmr.2187

[63] Li, C. et al. PLAGL1 overexpression induces cytoplasmic DNA accumulation that triggers cGAS/STING activation. *J. Cell Mol. Med.* **28**, e70130 (2024). 39365284 10.1111/jcmm.70130 PMC11451391

[64] Madisen, L. et al. A robust and high-throughput Cre reporting and characterization system for the whole mouse brain. *Nat. Neurosci.* **13**, 133–140 (2010). 20023653 10.1038/nn.2467 PMC2840225

[65] Zhang, J. et al. Occlusal effects on longitudinal bone alterations of the temporomandibular joint. *J. Dent. Res.* **92**, 253–259 (2013). 23340211 10.1177/0022034512473482 PMC6728563

[66] Ashburner, M. et al. Gene ontology: tool for the unification of biology. The Gene Ontology Consortium. *Nat. Genet.* **25**, 25–29 (2000). 10802651 10.1038/75556 PMC3037419

[67] Fabregat, A. et al. The Reactome Pathway Knowledgebase. *Nucleic Acids Res.* **46**, D649–d655 (2018). 29145629 10.1093/nar/gkx1132 PMC5753187

[68] Croft, D. et al. Reactome: a database of reactions, pathways and biological processes. *Nucleic Acids Res.* **39**, D691–D697 (2011). 21067998 10.1093/nar/gkq1018 PMC3013646

[69] Szklarczyk, D. et al. STRING v10: protein-protein interaction networks, integrated over the tree of life. *Nucleic Acids Res.* **43**, D447–D452 (2015). 25352553 10.1093/nar/gku1003 PMC4383874

[70] Shannon, P. et al. Cytoscape: a software environment for integrated models of biomolecular interaction networks. *Genome Res.* **13**, 2498–2504 (2003). 14597658 10.1101/gr.1239303 PMC403769

