# Supplementary material for: PLAGL1-IGF2 axis regulates osteogenesis of postnatal condyle development
Source: Int J Oral Sci. 2025 Sep 25;17:65. doi: 10.1038/s41368-025-00386-4 (PMC12462451; doi:10.1038/s41368-025-00386-4)
Supplement: Supplementary file 1 — Supplementary data [file 41368_2025_386_MOESM1_ESM.docx]

**PLAGL1-IGF2 axis regulates osteogenesis of postnatal condyle development**

Jinrui Sun^1,3,4,5^, Jingyi Xu^1,3,4,5^, Yue Xu^1,3,4,5^, Yili Liu^1,3,4^, Enhui Yao^1,3,4^, Jiahui Du^1,2,3,4#^, Xinquan Jiang^1,2,3,4#^

1 Department of Prosthodontics, Shanghai Ninth People’s Hospital, Shanghai Jiao Tong University School of Medicine, Shanghai, China.

2 Shanghai Stomatological Hospital & School of Stomatology, Fudan University, Shanghai

3 College of Stomatology, Shanghai Jiao Tong University, Shanghai, China.

4 National Center for Stomatology, National Clinical Research Center for Oral Diseases, Shanghai Key Laboratory of Stomatology, Shanghai Research Institute of Stomatology, Shanghai Engineering Research Center of Advanced Dental Technology and Materials, Shanghai, China.

5 These authors contributed equally: Jinrui Sun, Jingyi Xu, and Yue Xu.

# Corresponding author

Jiahui Du, Ninth People’s Hospital, School of Medicine, Shanghai Jiao Tong University, 639 Zhizaoju Road, Shanghai, 200011, China. Phone: +86 18321904501 Email: [jiahuidu2010@126.com](mailto:jiahuidu2010@126.com).

Xinquan Jiang, Ninth People’s Hospital, School of Medicine, Shanghai Jiao Tong University, 639 Zhizaoju Road, Shanghai, 200011, China. Phone: +86 13916918511. Email: [xinquanjiang@aliyun.com](mailto:xinquanjiang@aliyun.com)

**Figure S1**

**Figure S1. *Plagl1* deficiency impairs intramembranous ossification during mandibular morphogenesis. a** Micro-CT reconstructions of mandibular morphology in control (yellow dashed outline) and *Wnt1-Cre;Plagl1^pat fl/+^* littermates at PN 28.5. The right panels show the magnified standardized circular region of interest (1 mm diameter) at the body-ramus junction (white dashed demarcation in the left panels). Scale bars, 500 μm**. b** Measurements of the ratio of bone volume to tissue volume (BV/TV), bone surface area to bone volume ratio (BS/BV), bone mineral density (BMD) and porosity at the mandibular body-ramus junction in control and *Wnt1-Cre;Plagl1^pat fl/+^* littermates at PN 28.5. *n* = 4 per group. Unpaired two-tailed t-test was used for statistical analysis. The results are presented as the mean ± standard deviation (SD). **P* < 0.05.

**Figure S2**

**Figure S2. *Plagl1* deficiency does not significantly affect osteoclast activity in the subchondral bone of mouse condyles. a, b** Representative TRAP staining **(a)** and quantitative analysis **(b)** of osteoclastic surface/ bone surface (Oc.S/BS) in the subchondral bone of control and *Wnt1-Cre;Plagl1^pat fl/+^* mice at PN 28.5. White dashed lines show the boundary of subchondral bone. *n* = 3 per group. Scale bars, 125 μm. Unpaired two-tailed t-test was used for statistical analysis. The results are presented as the mean ± standard deviation (SD). ns, no significant difference.

| **Gene** | **Forward sequence** | **Reverse sequence** |
| --- | --- | --- |
| β-actin | GGCTGTATTCCCCTCCATCG | CCAGTTGGTAACAATGCCATGT |
| *Col1α1* | GCCTTGGAGGAAACTTTGCTT | GCACGGAAACTCCAGCTGAT |
| *Runx2* | GACTGTGGTTACCGTCATGGC | ACTTGGTTTTTCATAACAGCGGA |
| *Sp7* | ATGGCGTCCTCTCTGCTTG | TGAAAGGTCAGCGTATGGCTT |
| *Plagl1* | ATTCAAGTGCTCGAAGGCTGAGTG | GTGGTCCTTCCGGTTGAATGTC |
| *Igf2* | GTGCTGCATCGCTGCTTAC | ACGTCCCTCTCGGACTTGG |
| *Dmp1* | AGTGAGTCATCAGAAGAAAGTCAAGC | CTATACTGGCCTCTGTCGTAGCC |
|  |  |  |

**Table S1. List of qPCR primers.**
